# Multifunctional NaLnF_4_@MOF-Ln Nanocomposites with Dual-Mode Luminescence for Drug Delivery and Cell Imaging

**DOI:** 10.3390/nano9091274

**Published:** 2019-09-06

**Authors:** Dan Wang, Chen Zhao, Guoyang Gao, Linna Xu, Guofeng Wang, Peifen Zhu

**Affiliations:** 1 Key Laboratory of Functional Inorganic Material Chemistry, Ministry of Education, School of Chemistry and Materials Science, Heilongjiang University, Harbin 150080, China; wangdan1529@163.com (D.W.); zhaochen1815@163.com (C.Z.); 20145268@s.hlju.edu.cn (G.G.); xln8579xln@163.com (L.X.); 2 Department of Physics and Engineering Physics, The University of Tulsa, Tulsa, OK 74104, USA

**Keywords:** metal–organic framework, stokes/anti-stokes emission, dual-mode luminescence, cell imaging, drug delivery

## Abstract

Multifunctional nanomaterials for bioprobe and drug carrier have drawn great attention for their applications in the early monitoring the progression and treatment of cancers. In this work, we have developed new multifunctional water-soluble NaLnF_4_@MOF-Ln nanocomposites with dual-mode luminescence, which is based on stokes luminescent mesoporous lanthanide metal–organic frameworks (MOFs-Y:Eu^3+^) and anti-stokes luminescent NaYF_4_:Tm^3+^/Yb^3+^ nanoparticles. The fluorescence mechanism and dynamics are investigated and the applications of these nanocomposites as bioprobes and drug carriers in the cancer imaging and treatment are explored. Our results demonstrate that these nanocomposites with the excellent two-color emission show great potential in drug delivery, cancer cell imaging, and treatment, which are attributed to the unique spatial structure and good biocompatibility characteristics of NaLnF_4_@MOF-Ln nanocomposites.

## 1. Introduction

Cancer is one of the most serious threats to human health, which causes millions of deaths every year [1,2,3]. Traditional cancer treatment models have failed to meet the needs of clinical treatment [4].

Multifunctional nanomaterials integrated with disease diagnosis and treatment have attracted tremendous attention [5]. Tremendous efforts have been devoted to developing emerging biomaterials for tumor diagnosis and therapy. For example, peptide-based nanomaterials, magnetic hybrid nanomaterials (MHNs), and silica-based nanoparticles have been studied by a large number of people as multifunctional nanomaterials for tumor imaging and therapy [6,7,8].

Particle-based delivery systems can control the levels of drugs and their lifetime in vivo, as compared to conventional drug delivery forms that are assimilated by long-term oral administration or injection to maintain a constant drug concentration. Meanwhile, particle-based delivery systems can improve pharmacokinetics through the sustained release of the drug. In addition, particle-based systems are capable of delivering drugs at sites of interest to avoid biological and/or metabolic disorders, and this can result in higher drug delivery efficiencies, lower doses, and reduced side effects. In recent decades, many controlled release drug carrier systems such as quantum dots, organic micelles, dendrimers, inorganic mesoporous silica, and metal nanoparticles have been widely used [9,10,11]. Nevertheless, the drawbacks of low carrying capacity, poor degradability, and other shortcomings prevent the further application of these materials [12]. Therefore, the development of a drug carrier system with high throughput and controlled release capability remains challenging [13,14]. Metal–organic frameworks (MOFs) have developed rapidly over the past 20 years for their unique features. This type of materials can be self-assembled directly by organic linkages and metal centers [15,16,17]. Rare-earth MOF (MOF-Ln) is a type of hybrid porous materials composed of lanthanide metal ion clusters and organic ligands through coordination bonds, which is an important branch of metal–organic framework materials [18,19,20,21]. The versatile functions of MOFs have been widely used in many fields such as heterogeneous catalysis, chemical sensors, proton conduction, gas separation, bioimaging, and drug delivery [22,23,24,25]. MOF-Ln with unique optical and magnetic properties can be exploited as a biological imaging probe for fluorescence imaging and magnetic resonance imaging [26,27]. In addition, MOF has already been severed as a successful drug delivery platform as a result of the regular pore structure, a large pore volume, and a high surface area, which can effectively capture drug molecules and achieve high drug loading [28,29]. Meanwhile, MOF-Ln with the same structure is often explored for its luminescence mechanism or studied as a drug carrier.

In order to develop multifunctional materials for cancer treatment, we have studied the drug delivery capabilities of MOF-Ln and the bioimaging function of MOF-Ln. However, it is well known that most of the MOFs-Ln are also promising stokes emission materials [30,31]. Stokes single-mode imaging is often affected by background fluorescence in practical applications [32]. In order to solve this issue, we introduced anti-stokes luminescent materials. It is worth mentioning that anti-stokes luminescent materials have the advantages of strong penetration ability of near-infrared radiation (easier to image in vivo), photobleaching, and high detection limit [33,34,35]. In addition, the anti-stokes luminescent intensity of Tm^3+^/Yb^3+^ co-doped NaYF_4_ nanoparticles is several orders of magnitude higher than that of CdSe/ZnS quantum dots [36]. Therefore, the anti-stokes luminescent nanomaterials as fluorescent bioprobes have great application prospects in the fields of bioassay and medical diagnosis [37].

**Scheme 1:**
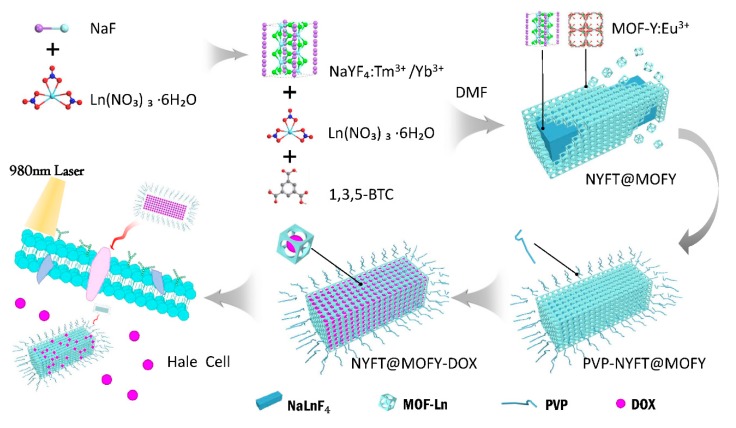
Schematic diagram of nanoparticle synthesis transmission, the release of anticancer drug DOX, and cellular labeling process.

In this work, we developed multifunctional materials with both disease treatment and cancer surveillance capabilities; water-soluble NaLnF_4_@MOF-Ln (Ln = Y, Tm, Yb, and Eu) nanocomposites were firstly synthesized with dual-mode luminescence by combining the anti-stokes luminescence of NaYF_4_:Tm^3+^/Yb^3+^ with the stokes luminescence of MOF-Y:Eu^3+^. The prepared water-soluble NaLnF_4_@MOF-Ln nanocomposites were successfully labeled with Hela cells, simultaneously carrying the anticancer drug doxorubicin hydrochloride (DOX) as a drug carrier for drug release in the simulated cancer cell environment (Scheme 1). The multifunctional NaLnF_4_@MOF-Ln nanocomposites with dual-mode luminescence and drug delivery function have great potential for cancer monitoring and treatment.

## 2. Materials and Methods

### 2.1. Synthesis of NaLnF_4_ Nanocrystals

To synthesize the NaLnF_4_ Nanocrystals, the 1.1 g of NaOH was dissolved in 4 mL of deionized water under stirring to obtain the NaOH solution. Then, 8 mL of ethanol and 18 mL of oleic acid were added in the above solution. After that, 2 mL of Ln(NO_3_)_3_ (Ln = 81.5% Y + 18% Yb + 0.5% Tm) aqueous solution (0.5 mol/L) was dropwise added to obtain solution A. Furthermore, 6 mmol of NaF was dissolved in 2 mL of deionized water to obtain NaF solution. The NaF solution was added to the solution A and the mixture was stirred for about 30 min. The milky white liquid was obtained and then it was transferred to 25 mL Teflon-lined autoclave and heated at 180 °C for 24 h. After heating, the system was cooled to room temperature and the products were washed with cyclohexane and ethanol. The final product was collected by centrifugation and dried in a vacuum oven at 60 °C.

### 2.2. Synthesis of NaLnF_4_@MOF-Ln Nanocomposites

The above products were firstly added to a mixture of Ln(NO_3_)_3_ (0.1 mmol Ln = 95% Y + 5% Eu) and deionized water (4 mL) and stirred for 30 min. A mixed solution of dimethylformamide (DMF) (8 mL) and 1, 3, 5-benzenetricarboxylic acid (20 mg) was added dropwise to the above solution, and vigorously stirred for 30 min. The final solution was transferred to a 25 mL Teflon-lined autoclave and heated at 60 °C for 24 h. Then, the systems were permitted to cool to room temperature. The final products were collected by centrifugation and dried under vacuum at 60 °C.

### 2.3. Preparation of PVP-Capped NaLnF_4_@MOF-Ln Nanocomposites

The prepared NaLnF_4_@MOF-Ln nanocrystals were dispersed in 2 mL of ethanol and sonicated for 5 min, and then 6 mL of an ethanol solution containing 0.8 g of polyvinylpyrrolidone (PVP) was added. After vigorous stirring, PVP-capped NaLnF_4_@MOF-Ln nanocrystals were obtained. This sample is named as (NaLnF_4_@MOF-Ln)@PVP.

### 2.4. Preparation of [(NaLnF_4_@MOF-Ln)@PVP]-DOX Nanocomposites

First, 10 mg of NaLnF_4_@MOF-Ln nanocomposites was dispersed in 4 mL of DOX aqueous solution (1 mg/mL), followed by ultrasonic treatment for 5 min. The DOX-loaded sample was continuously stirred in the dark for 24 h and collected by centrifugation.

### 2.5. Drug Storage/Delivery Studies

The NaLnF_4_@MOF-Ln-DOX was gently shaken with 2 mL of phosphate-buffered saline (PBS, pH = 7.4 and 5.0) at 37 °C to test drug release properties. At each time point, the PBS was removed and replaced with an equal volume of fresh PBS. Absorption spectra of DOX containing PBS were measured by using an ultraviolet-visible spectrophotometer. The concentration of DOX in PBS was then determined by using the standard DOX concentration-absorption curve. The total amount of released DOX was obtained by adding up all the released DOX at different time points.

### 2.6. In Vitro Cytotoxicity Assays of NaLnF_4_@MOF-Ln Nanocomposites

The in vitro cytotoxicity of NaLnF_4_@MOF-Ln nanocomposites was assessed by MTT assay for human cervical cancer HeLa cells. The cells were seeded in 96-well plates and cultured overnight at 37 °C in a 5% CO_2_ incubator. Then, they were incubated with different concentrations of NaLnF_4_@MOF-Ln nanocomposites for 24 h. The concentration of the nanocrystals was 10, 20, 40, 80, and 160 μg·mL^−1^, respectively. After the incubation, the cell culture medium containing NaLnF_4_@MOF-Ln nanocomposites was removed, and MTT solution (20 μL) was added in each cell. These cells were incubated for another 4 h. After removing the MTT, the wells were washed with PBS, and intracellular crystals were extracted with 150 μL of dimethyl sulfoxide (DMSO). After shaking the plate for 10 min, the absorbance of each well was measured by using a bio-tech Cytation 3 cell imaging multimode reader. Cell viability was calculated from the average obtained.

### 2.7. Materials Characterization

The crystal structure was analyzed by X-ray powder diffraction (XRD) patterns obtained by Bruker D8 Advance diffractometer by using Cu Kα radiation (λ = 1.5406 Å, 40 kV, 40 mA). The size and morphology of the products were investigated by transmission electron microscopy (TEM, JEOL, JEM-2100) and scanning electron microscopy (SEM). Thermogravimetry (TG) measurements were carried out on a thermal analyzer (TGA-7, Perkin-Elmer, Canton, MA, USA). Fourier transform infrared (FTIR) spectra were recorded at room temperature with a Perkin-Elmer Spectrumone FTIR spectrometer. The stokes luminescence spectra were recorded with a Hitachi F-4600 fluorescence spectrophotometer at room temperature (2.5 nm for spectral resolution (FWHM) of the spectrophotometer and 700 V for PMT voltage). The anti-stokes luminescence spectra were recorded using a Hitachi F-4600 fluorescence spectrophotometer with an adjustable laser (980 nm) as the excitation source with a fiber-optic accessory. For comparison of the luminescence properties of different samples, the luminescence spectra were measured with the same instrument parameters (2.5 nm for spectral resolution (FWHM) of the spectrophotometer and 400 V for PMT voltage). Fluorescence kinetics test used OPO pulsed laser as an excitation source.

### 2.8. Anti-Stokes Luminescent Imaging of NaLnF_4_@MOF-Ln Nanocomposites

An inverted fluorescence microscope (Olympus IX71, Tokyo, Japan) and a 980 nm infrared laser (CNI, MDL-H-980/5000mW, Changchun, China) were used to illuminate the sample. HeLa cells were seeded in glass-bottomed dishes and incubated overnight. After the cells were incubated with NaLnF_4_@MOF-Ln nanocomposites for 4 h at 37 °C, all cells were washed with PBS solution. The cells were imaged using a fluorescence microscope and the cells were illuminated with a 980 nm infrared laser with an output power of 250 mW.

## 3. Results and Discussion

### 3.1. Characterization of NaLnF_4_@MOF-Ln Nanocomposites

**Figure 1:**
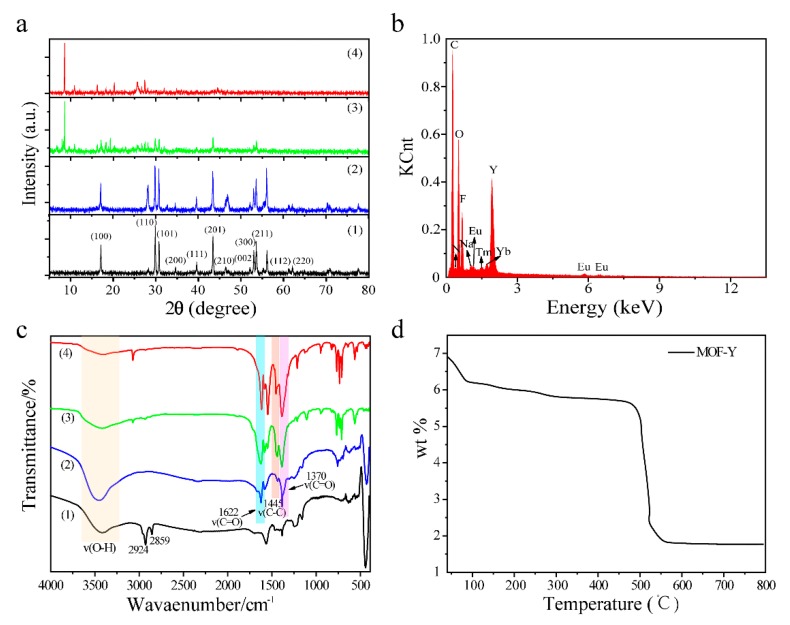
(**a**) XRD patterns of (1) NaYF_4_:Tm^3+^/Yb^3+^, (2) NYFT@MOFY-10 nanocomposites, (3) NYFT@MOFY-1 nanocomposites, (4) MOF-Y:Eu^3+^; (**b**) energy dispersive X-ray analysis (EDX) analysis of the NYFT@MOFY-1 nanocomposites; (**c**) FT-IR spectra of (1) NaYF_4_:Tm^3+^/Yb^3+^, (2) NYFT@MOFY-10, (3) NYFT@MOFY-1, and (4) MOF-Y:Eu^3+^; (**d**) thermogravimetry (TG) curves of MOF-Y:Eu^3+^.

The different abbreviations of NaLnF_4_@MOF-Ln and the synthetic conditions are detailed in Table 1. Figure 1a shows the XRD patterns of the prepared NaYF_4_:Tm^3+^/Yb^3+^, MOF-Y:Eu^3+^, and NaYF_4_:Tm^3+^/Yb^3+^@MOF-Y:Eu^3+^ nanocomposites. The hexagonal-phase NaYF_4_:Tm^3+^/Yb^3+^ (JCPDS No. 28-1192) nanocrystals was obtained in Figure 1a curve (1). The curve (4) in Figure 1a is the XRD pattern of MOF-Y:Eu^3+^, which completely matches the reported XRD pattern of MOF-Y [38]. The characteristic peaks of MOF-Y:Eu^3+^ became more obvious with increasing the ratio of MOF-Y:Eu^3+^ to NaYF_4_:Tm^3+^/Yb^3+^ in NaLnF_4_@MOF-Ln composites as shown in Figure 1a curve (2) and curve (3).

The composition of the NaYF_4_:Tm^3+^/Yb^3+^@MOF-Y:Eu^3+^ nanocomposites was determined by using the energy dispersive X-ray analysis (EDX). Figure 1b displays that the elements in the NYFT@MOFY-1 nanocomposites are C, O, N, Na, F, Eu, Y, Yb, and Tm, which indicates that NaYF_4_:Tm^3+^/Yb^3+^ and MOF-Y:Eu^3+^ coexist in the NYFT@MOFY-1 nanocomposites.

The Fourier transform infrared (FT-IR) spectra of NaYF_4_:Tm^3+^/Yb^3+^, MOF-Y:Eu^3+^, and NaYF_4_:Tm^3+^/Yb^3+^@MOF-Y:Eu^3+^ nanocomposites are shown in Figure 1c. In the FT-IR spectrum of NaYF_4_: Tm^3+^/Yb^3+^, the two peaks at 2924 and 2859 cm^−1^ are the asymmetry and symmetric tensile vibration of methane in oleic acid molecules, respectively. In the FT-IR spectrum of MOF-Y:Eu^3+^, the three peaks at 1370 cm^−1^, 1622 cm^−1^, and 1455 cm^−1^ are produced by the stretching vibration of the C=O bond and the C=C bond of MOF-Y:Eu^3+^, respectively. The peaks at 824 and 710 cm^−1^ are characteristics of a 1, 3, 5-benzenetricarboxylic acid (1, 3, 5-BTC) of the benzene ring. These results indicate that the constituent part of MOF contains a carboxyl group and a basic 1,3,5-BTC backbone, of which 1,3,5-BTC is deprotonated. In the FT-IR spectra of NYFT@MOFY-10 and NYFT@MOFY-1, the broadband located at 3410 cm^−1^ is assigned to the O–H vibration, meaning the existence of free water and bound water [39]. The peaks at 1622 and 1370 cm^−1^ are from carboxyl groups of asymmetric and symmetric vibrations, indicating the formation of NaYF_4_:Tm^3+^/Yb^3+^@MOF-Y:Eu^3+^ Nanocomposites.

Figure 1d is the thermogravimetric (TG) curve of the MOF-Y:Eu^3+^. In general, the curve has four stages of thermal weight loss, and the biggest weight loss occurs in the final stage. The first and the second weight-loss stages represent the evaporation of the solvent in the MOF-Y:Eu^3+^ channel. The weight loss of the third stage (detection point at 315.5 °C) is the result of the initial thermolysis of the ligand. The total weight loss of these three stages is 18 wt%. In the temperature range from 471.2 to 563.3 °C, the thermal weight-loss ratio changed from 18 wt% to 64.3 wt%. This mutation is due to collapse of the frame structure at high temperatures. Thus, the MOF-Y:Eu^3+^ is stable up to ~471 °C. This indicates that the MOF-Y:Eu^3+^ has good thermal stability.

**Figure 2:**
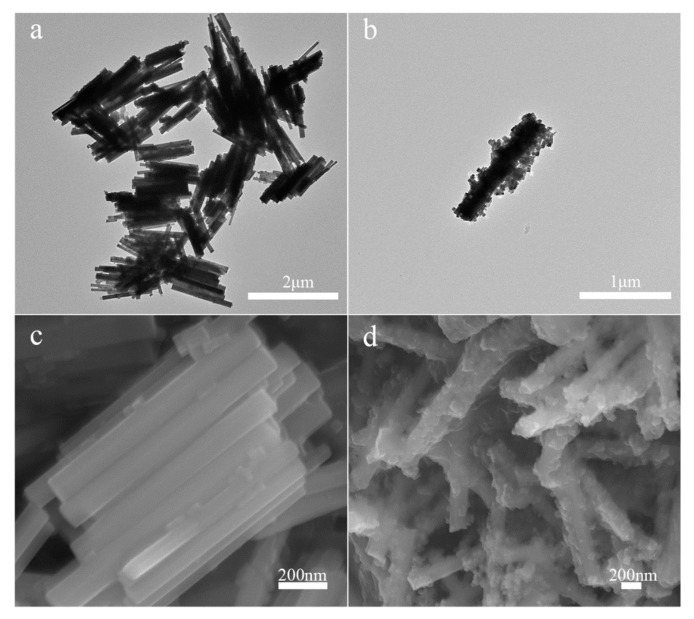
The TEM images of (**a**) NaYF_4_:Tm^3+^/Yb^3+^ and (**b**) NYFT@MOFY-1 nanocomposites. The SEM images of (**c**) NaYF_4_:Tm^3+^/Yb^3+^ and (**d**) NYFT@MOFY-1 nanocomposites.

**Figure 3:**
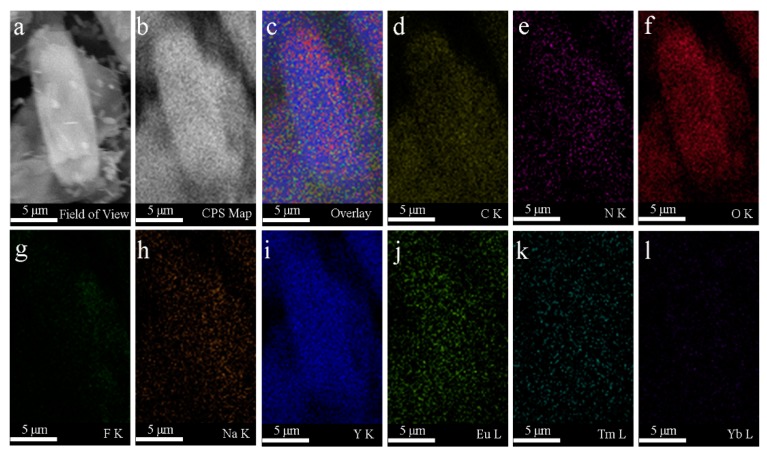
(**a**) SEM image of the NYFT@MOFY-1 sample. (**b**–**l**) SEM element mapping of the corresponding NYFT@MOFY-1 composites, C, N, O, F, Na, Y, Eu, Tm, and Yb, respectively.

Transmission electron microscopy (TEM) and scanning electron microscopy (SEM) were used to characterize the morphology. As shown in Figure 2a, the NaYF_4_:Tm^3+^/Yb^3+^ is stick-shaped. Figure 2b shows the TEM image of the NYFT@MOFY-1 nanocomposites, indicating that the MOF-Y:Eu^3+^ is attached to NaYF_4_: Tm^3+^/Yb^3+^. The SEM images of the NaYF_4_: Tm^3+^/Yb^3+^ and NYFT@MOFY-1 nanocomposites are shown in Figure 2c,d, which is consistent with the TEM results mentioned above. To further reveal structure in NYFT@MOFY-1 heterostructures, SEM elemental mapping analysis is carried out (Figure 3) through the particle mapping test. It is not difficult to find that the particles are composed of NaYF_4_:Tm^3+^/Yb^3+^ and MOF-Y:Eu^3+^. Structure analysis indicates that NYFT@MOFY-1 heterostructures have uniform stick shape.

### 3.2. Luminescence Performance of NaYF_4_:Tm^3+^/Yb^3+^@MOF-Y:Eu^3+^ Nanocomposites

**Figure 4:**
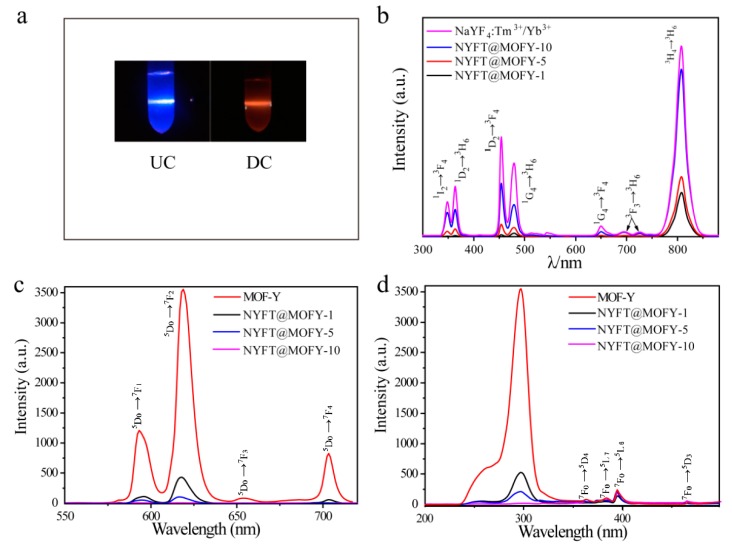
(**a**) Photographs of anti-stokes luminescence under 980-nm laser excitation and stokes luminescence of NYFT@MOFY-1 nanocomposites 290-nm light excitation; (**b**) anti-stokes emission spectra of NaYF_4_:Tm^3+^/Yb^3+^, NYFT@MOFY-10, NYFT@MOFY-5, and NYFT@MOFY-1 under 980-nm excitation; (**c**) stokes emission spectra of MOF-Y, NYFT@MOFY-1, NYFT@MOFY-5, and NYFT@MOFY-10 excited at 290 nm; and (**d**) stokes excitation spectra of MOF-Y, NYFT@MOFY-1, NYFT@MOFY-5, and NYFT@MOFY-10 monitored at 618 nm.

Figure 4a is the images showing the anti-stokes and stokes luminescence of NYFT@MOFY-1 nanocomposites. The bright blue emission was obtained under 980-nm light illumination and the dark red light was observed under 290-nm light excitation. This indicates that NaYF_4_:Tm^3+^/Yb^3+^@MOF-Y:Eu^3+^ nanocomposites possess dual-mode luminescence properties. Their dual luminescence properties were also quantified by measuring anti-stokes and stokes luminescence spectra. Figure 4b shows the anti-stokes emission spectra of NaYF**_4_**: Tm^3+^/Yb^3+^ and NaYF**_4_**: Tm^3+^/Yb^3+^@MOF-Y:Eu^3+^ nanocomposites. The two blue emission peaks at 450 and 474 nm correspond to the ^1^D_2_→^3^F_4_ and ^1^G_4_→^3^H_6_ transition, respectively. The anti-stokes emission peaks at 347, 363, 642, 689, and 795 nm correspond to the ^1^I_6_→^3^F_4_, ^1^D_2_→^3^H_6_, ^1^G_4_→^3^F_4_, ^3^F_3_→^3^H_6_, and ^3^H_4_→^3^H_6_ transitions, respectively. In addition, the luminescence intensity of NYFT@MOF-Y decreases with increasing the ratio of MOF-Y:Eu^3+^ to NaYF_4_: Tm^3+^/Yb^3+^, which is expected because the amount of anti-stokes luminescence center decreases.

The emission spectra of the NaYF**_4_**: Tm^3+^/Yb^3+^@MOF-Y:Eu^3+^ excited at 290 nm are shown in Figure 4c. The transitions from Eu^3+^ were observed (579 nm, from ^5^D_0_ to ^7^F_1_; 590 nm, from ^5^D_0_ to ^7^F_1_; 616 nm, from ^5^D_0_ to ^7^F_2_; 652 nm, from ^5^D_0_ to ^7^F_3_; 701 nm, from ^5^D_0_ to ^7^F_4_). The intensity of stokes luminescence increases with increasing of the ratio of MOF-Y:Eu^3+^ to NaYF_4_: Tm^3+^/Yb^3+^ and maximum intensity is obtained from MOF-Y:Eu^3+^, which is consistent with the trend of anti-stokes luminescence intensity. Figure 4d shows the excitation spectra of the NaYF**_4_**: Tm^3+^/Yb^3+^@MOF-Y:Eu^3+^ nanocomposites by monitoring the emission at the 618 nm. The broad excitation band at 220–350 nm is primarily ascribed to the absorption of the MOF-Y matrix, and the energy is transferred to the excitation levels of the Eu^3+^ ions, thereby producing a sharp and intense light emission. In addition, the characteristic excitation peaks of Eu^3+^ ions centered at 361, 381, 393, 413, and 463 nm correspond to the ^7^F_0_→^5^D_4_, ^7^F_0_→^5^L_7_, ^7^F_0_→^5^L_6_, and ^7^F_0_→^5^D_3_ transitions, respectively. Note that emission from Eu is much more effective by exciting the MOF-Y matrix than of Eu^3+^. Thus, the MOFs are great host materials for luminescence centers. The stokes emission spectra of NYFT@MOFY-1 excited by different wavelengths of light are shown in Figure S1a and excitation wavelength-dependent intensity is observed, which is consistent with the excitation spectrum shown in Figure 4d. The excitation spectra by monitoring emissions at different wavelengths are also shown in Figure S1b and, as expected, it also shows wavelength-dependent features. Thus, the excitation wavelength has a significant effect on the intensity of emission.

**Figure 5:**
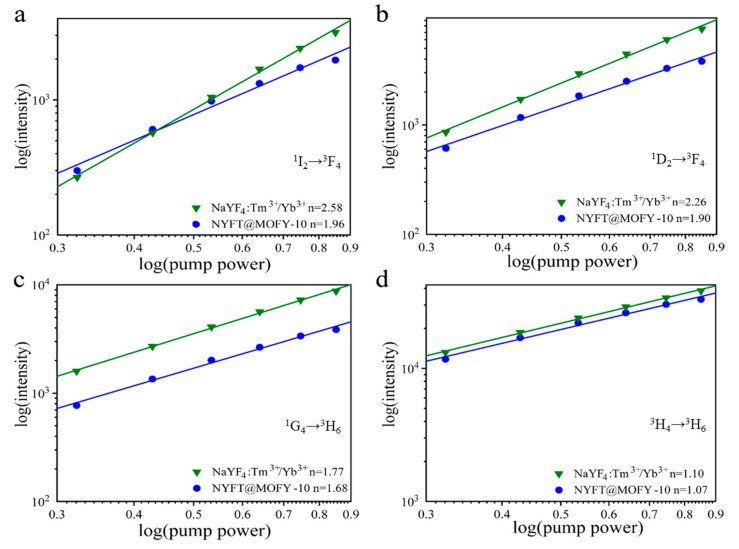
Plots (log–log) of the emission intensity versus pump power density for different samples at (**a**) ^1^I_2_→^3^F_4_ (Tm^3+^), (**b**) ^1^D_2_→^3^F_4_ (Tm^3+^), (**c**) ^1^G_4_→^3^H_6_ (Tm^3+^), and (**d**) ^3^H_4_→^3^H_6_ (Tm^3+^).

To further investigate the anti-stokes mechanism of NaYF_4_: Tm^3+^/Yb^3+^ and NaYF_4_:Tm^3+^/Yb^3+^@MOF-Y:Eu^3+^ nanocomposites, the UV (^1^I_2_→^3^F_4_), visible (^1^D_2_→^3^F_4_, ^1^G_4_→^3^H_6_), and IR (^3^H_4_→^3^H_6_) emissions were measured as a function of excitation power, as shown in Figure 5. According to the relation between intensity and pump power, $I\;\infty \;P^{n}$, the emission intensity is proportional to the certain power (*n*) of the infrared excitation intensity, and the integer n is the number of photons absorbed per upconverted photon emitted [40]. For the UV emission, the values of *n* values at ^1^I_2_→^3^F_4_ transition of NaYF_4_:Tm^3+^/Yb^3+^ and NYFT@MOFY-10 are determined to be 2.58 and 1.96 (Figure 5a), respectively, indicating two-photon processes. For visible emission at the transitions of ^1^D_2_→^3^F_4_ of NaYF_4_: Tm^3+^/Yb^3+^ and NYFT@MOFY-10 (Figure 5b), the values of n are determined to be 2.26 and 1.9, which are attributed to two-photon processes. For the transitions of ^1^G_4_→^3^H_6_ of NaYF_4_: Tm^3+^/Yb^3+^ and NYFT@MOFY-10, the values of n are determined to be values of 1.77 and 1.68 (Figure 5c), respectively, which is originated from two-photon processes. For IR emission from ^3^H_4_→^3^H_6_ (Tm^3+^), the values of n are determined to be 1.10 and 1.07 for NaYF_4_:Tm^3+^/Yb^3+^ and NYFT@MOFY-10, respectively, as shown in Figure 5d, indicating one-photon processes. Note that the values of n become slight smaller after forming NYFT@MOFY nanocomposites compared to that of NaYF_4_:Tm^3+^/Yb^3+^. This might be attributed to the weak energy transfer from NaYF_4_:Tm^3+^/Yb^3+^ to MOF-Y:Eu^3+^ as we can see from the slight overlap of excitation spectra of MOF-Y:Eu^3+^ and emission spectra of NaYF_4_:Tm^3+^/Yb^3+^. The stronger overlap leads to more decrease of the n (Figure 5a,b) and the weaker overlap results in less decrease of n (Figure 5c,d). This further proves that NYFT@MOFY nanocomposites were formed.

**Figure 6:**
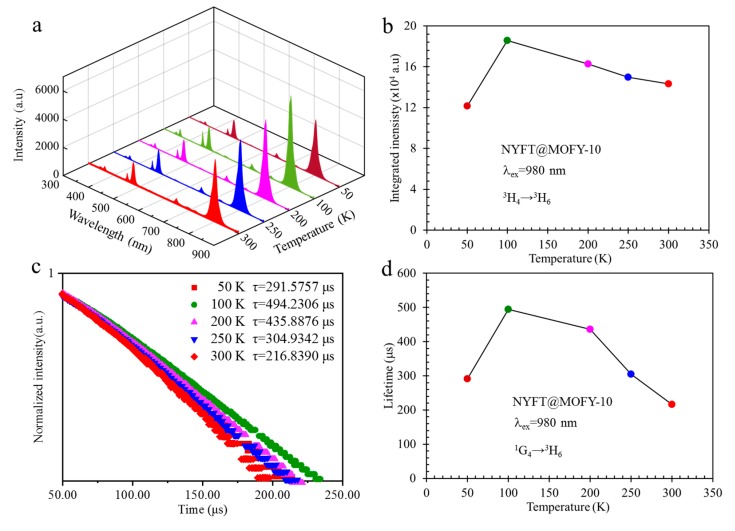
(**a**) The fluorescence spectra of NYFT@MOFY-10 nanocomposites at different temperatures under 980-nm light excitation, (**b**) the intensity of the transition of ^3^H_4_→^3^H_6_ as a function of temperature, (**c**) fluorescence decay curves for the transition of ^1^G_4_→^3^H_6_ at different temperatures, and (**d**) the lifetime as a function of temperature.

The effect of temperature on the anti-stokes fluorescence of NYFT@MOFY-10 nanocomposites was investigated by measuring photoluminescence spectra and lifetimes at various temperatures from 50 K to 300 K under 980-nm light excitation. The fluorescence intensity shows temperature dependence for some transitions as shown in Figure 6a. The changes of intensity with temperature for the transition of ^3^H_4_→^3^H_6_ is plotted in Figure 6b. The intensity gradually increases with decreasing the temperature from 300 K to 100 K and the maximum intensity is obtained at a temperature of 100 K. The intensity starts to decrease when the temperature is further decreased from 100 K to 50 K. This is attributed to the energy transfer and thermal quenching effect as reported in the previous work [41,42]. The Yb^3+^ absorbed the photons under 980-nm light excitation and electrons move from ^2^F_7/2_ to ^2^F_5/2_ [43]. Then, the energy was transferred from Yb^3+^ to Tm^3+^ to emit light. Thus, the fluorescence intensity is determined by absorption efficiency, energy-transfer efficiency, and radiative recombination efficiency. In the crystal field, the ^2^F_5/2_ energy level is split into ^2^F_5/2_|0> and ^2^F_5/2_|1> energy levels. The energy transfer from ^2^F_5/2_|1> to Tm^3+^ is more efficient [41]. Based on Boltzmann distribution, there are more electrons occupying ^2^F_5/2_|1> energy level at the higher temperatures. Therefore, the energy-transfer efficiency is higher at higher temperatures, which leads to high fluorescence intensity. On the other hand, non-radiative relaxation processes increase with increasing the temperature, which results in lower radiative recombination efficiency and lower fluorescence intensity. Highest intensity is obtained at a tradeoff between these two factors. Thus, the intensity increases with the temperature first and then decreases.

The fluorescence decay curves were measured by monitoring the 480-nm emission under 980-nm laser light excitation at different temperatures as shown in Figure 6c. The highest intensity is obtained at the temperature of 100 K, which is consistent with our temperature-dependent fluorescence spectrum measurement as shown in Figure 6a. The intensity decreases with the time in general, however, the decreasing rate is different at different temperatures. To quantitatively characterize the fluorescence dynamics, the fluorescence intensity was fitted using *I* = *e*^−*t*/*τ*^ (*I*_0_ is the intensity at *t* = 0; *I* is the intensity at time *t*; and *τ* is the fluorescence lifetime) and the lifetimes (*τ*) were obtained. We find that the fluorescence lifetime under 980-nm light excitation increases with temperature and then decreases as shown in Figure 6d, which is consistent with results obtained from temperature-dependent fluorescence measurement. This is also in a good agreement with the previous report [44,45]. The slow decay demonstrates the pure ground-state absorption/energy transfer anti-stokes process (GSA/ETU), which is much slower than the direct excitation of Tm^3+^ [45].

### 3.3. Drug Release from NaYF_4_:Tm^3+^/Yb^3+^@MOF-Y:Eu^3+^ Nanocomposites

**Figure 7:**
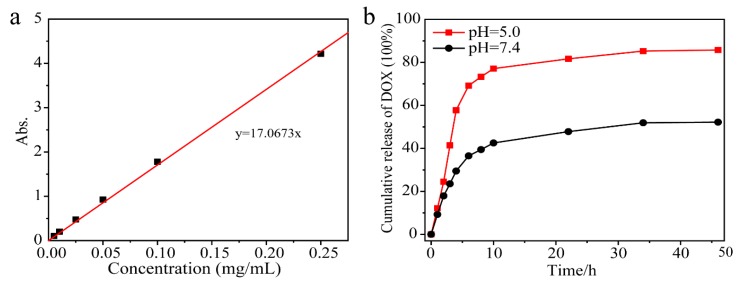
(**a**) Standard curve of DOX concentration and absorption, (**b**) drug release profiles for NYFT@MOFY in PBS buffer solution at pH = 5.0 and in PBS buffer solution at pH = 7.4.

Because the microenvironment of tumor intracellular lysosomes and extracellular tissues are acidic, carriers or drugs with pH-stimulated responses are ideal release systems. The release properties of the materials were investigated by selecting PBS solution buffer solutions with pH values of 7.4 and 5.0, respectively [46,47]. Figure 7 shows the release profile of the drug assembly at pH = 5.0 and pH = 7.4. The normal physiological environments were simulated in neutral PBS (pH 7.4) and DOX was particularly slow to release from NaYF_4_:Tm^3+^/Yb^3+^@MOF-Y:Eu^3+^ nanocomposites. The maximum accumulative release of DOX is approximately 52.2%. However, by simulating the intracellular environment of tumor cells in PBS pH of 5.0, the release rate of DOX is speeding up. The accumulative release of DOX can achieve approximately 85.8%. DOX was shown to be more efficiently transported into the cells by NaYF_4_:Tm^3+^/Yb^3+^@MOF-Y:Eu^3+^ nanocomposites platform and can release slowly in cancer cells, rather than used alone. Therefore, it can be concluded that NaYF_4_:Tm^3+^/Yb^3+^@MOF-Y:Eu^3+^ nanocomposites have promising drug carrier potential.

### 3.4. Cell Imaging

**Figure 8:**
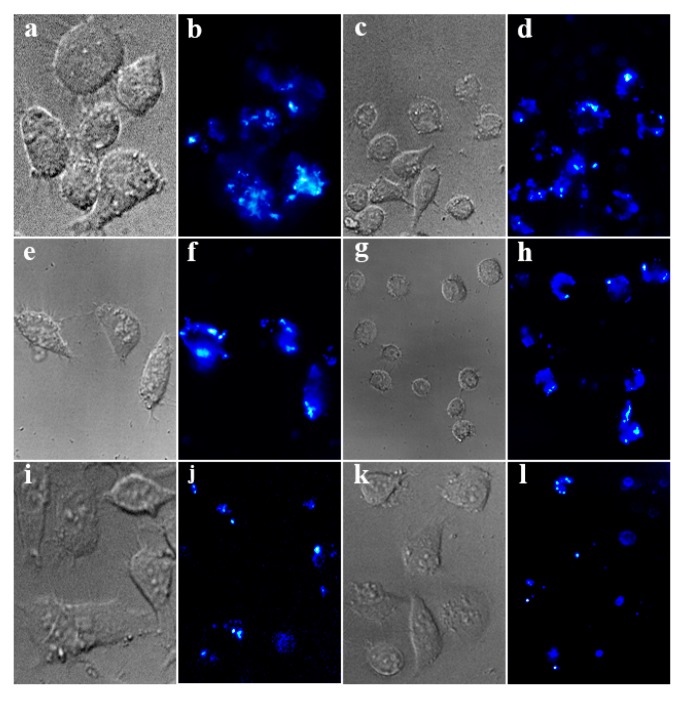
Hela cells were incubated with (**a**–**d**) NaYF_4_:Tm^3+^/Yb^3+^@MOF-Y:Eu^3+^ supernatant, (**e**–**h**) NaYF_4_:Tm^3+^/Yb^3+^@MOF-Y:Eu^3+^ solution, and (**i**–**l**) NaGdF_4_:Tm^3+^/Yb^3+^@MOF-Gd:Eu^3+^ solution; the fluorescence microscopy images were irradiated with a laser at 980 nm.

We modified NaYF_4_:Tm^3+^/Yb^3+^@MOF-Y:Eu^3+^ nanocomposites surface with PVP to promote the cellular uptake of the NaYF_4_:Tm^3+^/Yb^3+^@MOF-Y:Eu^3+^ nanocomposites. NaYF_4_:Tm^3+^/Yb^3+^@MOF-Y:Eu^3+^ nanocomposites’ low concentration in a shorter incubation time results in high intensity brightness on labeled cells. According to previous work, the fluorescent signal has been confirmed to be tracked in vitro for a long time [48,49]. Therefore, as a fluorescent probe, anti-stokes luminescent nanomaterials have developed a new and potential monitoring tool for early-stage tumor diagnosis. We incubated the supernatant of NaYF_4_:Tm^3+^/Yb^3+^@MOF-Y:Eu^3+^ nanocomposites and an aqueous solution with Hela cells for 4 h for cell imaging. The use of a fluorescence microscope with a 980-nm source to obtain a fluorescent labeling image is shown in Figure 8. We observe that the light-emitting cell turnover labeling on the cell and NaYF_4_:Tm^3+^/Yb^3+^@MOF-Y:Eu^3+^ nanocomposites under bright field coincides. This result indicates that Hela is efficiently labeled by NaYF_4_:Tm^3+^/Yb^3+^@MOF-Y:Eu^3+^ nanocomposites and it is found mainly in the cytoplasm and nanoparticles perinuclear region as shown in Figure 8b,d,f,h. Compared to NaGdF_4_:Tm^3+^/Yb^3+^@MOF-Gd:Eu^3+^ nanocomposites (Figure 8i–l), NaYF_4_:Tm^3+^/Yb^3+^@MOF-Y:Eu^3+^ nanocomposites have higher brightness and can label cancer cells more completely. It indicates that cellular uptake of NaYF_4_:Tm^3+^/Yb^3+^@MOF-Y:Eu^3+^ nanocomposites may be performed by energy-dependent endocytosis.

### 3.5. In Vitro Cell Viability Test

The in vitro cytotoxicity of NaLnF_4_@MOF-Ln nanocomposites was assessed by MTT assay. Hela cells treated with NaYF_4_:Tm^3+^/Yb^3+^@MOF-Y:Eu^3+^ showed high cell viability even in the sample concentration up to 160 µg·mL^−1^ (Figure S2). Therefore, it indicates that the nanocomposites have good biocompatibility. The results demonstrate that NaYF_4_:Tm^3+^/Yb^3+^@MOF-Y:Eu^3+^ nanocomposites can be used as fluorescent probes to detect cancer cells, which can be applied to cell imaging.

## 4. Conclusions

In this work, the dual-mode luminescent multi-functional nanocarriers, NaLnF_4_@MOF-Ln nanocomposites, were successfully prepared for the first time. Anti-stokes luminescence NaYF_4_:Tm^3+^/Yb^3+^ nanoparticles were synthesized by the hydrothermal method. The NaLnF_4_@MOF-Ln nanocomposites were obtained by coating MOF-Y:Eu^3+^ on the surface of NaYF_4_:Tm^3+^/Yb^3+^. The PVP-capped NaLnF_4_@MOF-Ln nanocomposites can be well dispersed in water and organic solvents. The NaLnF_4_@MOF-Ln nanocomposites obtained as above showed a unique anti-stokes blue fluorescence and stokes red fluorescence under laser excitation at a specific wavelength. Compared to the virulence of semiconductor quantum dots and the quenching and bleaching of fluorescent organic molecules, NaLnF_4_@MOF-Ln nanocomposites offer fluorescence imaging capabilities as a vivo bioprobe. Furthermore, cytotoxicity is a pivotal element in assessing the prospects of biomedical applications of nanomaterials. Non-toxic NaLnF_4_@MOF-Ln nanocomposites are suitable for serving as pharmaceutical carriers. In two different pH phosphate-buffered saline (PBS) environments, the drug (DOX) loading and release behavior of (NaLnF_4_@MOF-Ln)@PVP showed pH dependence. In the acidic microenvironment of simulated cancer cells, (NaLnF_4_@MOF-Ln)-DOX can achieve slow and efficient release. It indicates the possibility of using NaLnF_4_@MOF-Ln as drug delivery systems for biological applications. Our findings show that these dual-mode luminescent NaLnF_4_@MOF-Ln multifunctional nanocomposites have potential in the integration of cancer therapy and diagnosis.

## Figures and Tables

**Table 1:** Abbreviations of NaLnF_4_@MOF-Ln studied in this work.

Name	NaYF_4_: Tm^3+^/Yb^3+^ (g)	MOF-Y: Eu^3+^ (g)	Weight Ratio
NYFT@MOFY-1	0.01	0.01	1:1
NYFT@MOFY-5	0.05	0.01	5:1
NYFT@MOFY-10	0.1	0.01	10:1

## Supplementary Materials

The following are available online at https://www.mdpi.com/2079-4991/9/9/1274/s1, Figure S1: (a) stokes emission spectra of NYFT@MOFY-1 excited at different wavelengths, (b) stokes excitation spectra of NYFT@MOFY-1 by monitoring emission at different wavelengths, Figure S2: In vitro cell viability was measured by MTT assay after incubation of Hela cancer cells at different concentrations with pure NYFT@MOFY-1 nanocomposites for 4 h.
