# Supplementary material for: Multifunctional NaLnF4@MOF-Ln Nanocomposites with Dual-Mode Luminescence for Drug Delivery and Cell Imaging
Source: Nanomaterials (Basel). 2019 Sep 6;9(9):1274. doi: 10.3390/nano9091274 (PMC6781070; doi:10.3390/nano9091274)
Supplement: Supplementary file 1 [file nanomaterials-09-01274-s001.pdf]

Article

Multifunctional $NaLnF_4$@MOF-Ln Nanocomposites with Dual-Mode Luminescence for Drug Delivery and Cell Imaging

Dan Wang [1], Chen Zhao[1], Guoyang Gao[1], Linna Xu[1], Guofeng Wang [1,*] and Peifen Zhu [2,*]

[1] Affiliation[1]Key Laboratory of Functional Inorganic Material Chemistry, Ministry of Education, School of Chemistry and Materials Science, Heilongjiang University, Harbin, 150080, China

[2] Department of Physics and Engineering Physics, The University of Tulsa, Tulsa, Oklahoma 74104, USA

* Correspondence: 2010070@hlju.edu.cn (G. W); peifen-zhu@utulsa.edu (P. Z); Tel.: +1-918-631-5125 (G. W)

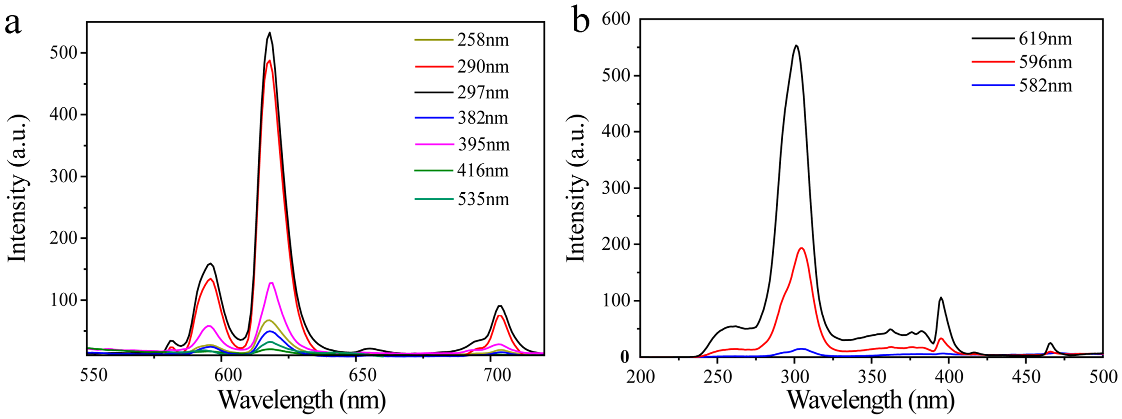

Figure S1 (a) DC emission spectra of NYFT@MOFY-1 excited at different wavelengths, (b) DC excitation spectra of NYFT@MOFY-1 by monitoring emission at different wavelengths.

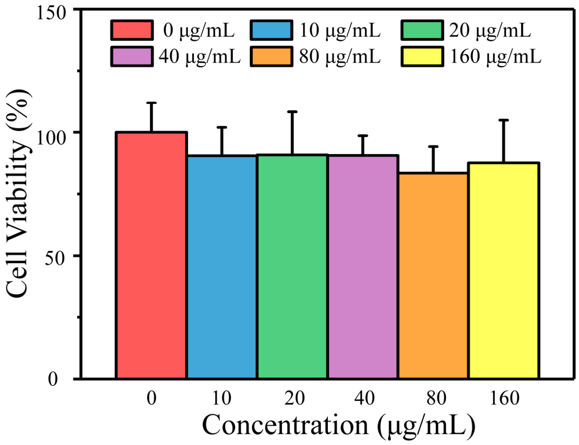

Figure S2. In vitro cell viability was measured by MTT assay after incubation of Hela cancer cells at different concentrations with pure NYFT@MOFY-1 nanocomposites for 4 h.
